# Bacterial Transcriptional Regulators: A Road Map for Functional, Structural, and Biophysical Characterization

**DOI:** 10.3390/ijms23042179

**Published:** 2022-02-16

**Authors:** Cristian M. Pis Diez, Maria Juliana Juncos, Matias Villarruel Dujovne, Daiana A. Capdevila

**Affiliations:** Fundación Instituto Leloir, Av. Patricias Argentinas 435, Buenos Aires C1405BWE, Argentina; cpisdiez@leloir.org.ar (C.M.P.D.); mjuncos@leloir.org.ar (M.J.J.); mvillarruel@leloir.org.ar (M.V.D.)

**Keywords:** bacteria, transcriptional regulators, structural biology, allosteric coupling

## Abstract

The different niches through which bacteria move during their life cycle require a fast response to the many environmental queues they encounter. The sensing of these stimuli and their correct response is driven primarily by transcriptional regulators. This kind of protein is involved in sensing a wide array of chemical species, a process that ultimately leads to the regulation of gene transcription. The allosteric-coupling mechanism of sensing and regulation is a central aspect of biological systems and has become an important field of research during the last decades. In this review, we summarize the state-of-the-art techniques applied to unravel these complex mechanisms. We introduce a roadmap that may serve for experimental design, depending on the answers we seek and the initial information we have about the system of study. We also provide information on databases containing available structural information on each family of transcriptional regulators. Finally, we discuss the recent results of research about the allosteric mechanisms of sensing and regulation involving many transcriptional regulators of interest, highlighting multipronged strategies and novel experimental techniques. The aim of the experiments discussed here was to provide a better understanding at a molecular level of how bacteria adapt to the different environmental threats they face.

## 1. Introduction

Bacteria have colonized essentially every type of niche, regardless of how harsh a particular environment might be. A single bacterium often moves through several niches during its life cycle; thus, a rapid response to the environmental changes and presence of stressor molecules is critical. For this to occur, bacteria must correctly sense and transduce various environmental signals by using complex signaling pathways that ultimately lead to the transcription of specific genes required for survival in each situation. This response is orchestrated by transcriptional regulators (TRs) that can function as single-component systems, as DNA-binding proteins with the ability to modulate function as a response to the presence of some specific chemical species [1], or as part of a more complex signaling pathway [2,3]. Recently, structural details of small molecule and metal ion sensing riboswitches have also appeared [4,5], making it clear that both protein and RNA have the level of specificity necessary to regulate transcription outcomes in response to small molecules. Generally, TRs have two domains or motifs—one that harnesses the residues involved in the specific interactions with the operator region of the DNA (namely, DNA-binding domain or site) and another one that is involved in inducer recognition (namely, regulatory domain or site). With some TRs, the regulatory domain might have more tasks beyond the modulation of the DNA-binding affinity, including ligand binding, protein–protein interaction, and enzymatic activity.

TRs can act as repressors or activators. Generally, the transcriptional repressor bound to DNA represses transcription by blocking the activity of RNA polymerase, and in the presence of the inducer, the TR dissociates from DNA and initiates transcription. On the contrary, transcriptional activators enhance transcription by binding to DNA, for example, by recruiting RNA polymerase for the activation of gene transcription [6].

The process by which the binding event at the regulatory domain influences DNA binding at the DNA-binding domain, and thus, the regulation over gene expression, is termed allostery. Allostery consists of a fundamental thermodynamic phenomenon in which the binding of one ligand influences the binding of a second ligand or activity at a physically distinct site. Allosteric communication between two ligand-binding sites in a protein is a central aspect of biological regulation. Identifying what physical features poise a certain conformational state for ligand binding remains an important goal in efforts to understand biological regulation.

The structural biology revolution experienced during the last two decades has served not only to provide invaluable experimental and predicted structural details of various states of proteins and nucleotide complexes [7,8,9] but has also made clear that proteins exist as dynamic conformational ensembles, interconverting between different conformations with varying energies [10,11,12,13]. This distribution of ensembles is not static, as the populations shift upon the formation of a covalent bond, noncovalent binding, temperature changes, or other phenomena [14]. The population shift is a consequence of a change in the relative stabilities of the conformations and is indeed the origin of the allosteric effect. In these ensemble models of protein function, allostery can be considered a change in the affinity for a ligand by the shift in the accessible conformations upon another ligand binding at a different location in the macromolecule [10,13,15,16] rather than a structural change between two static conformations. The experimental results that support these models in the context of allostery in TRs are discussed in Section 5 of this review.

TRs are classified in protein families based on sequence similarities, particularly in the DNA-binding region. Moreover, members of the same family are characterized by a certain degree of conservation in the overall molecular architecture and structural motifs. The chemical species eliciting a transcriptional response may be metal ions, metalloids, small organic molecules, or even lipids. To some extent, the magnitude of the structural changes derived from the inducer binding event in a particular TR can be predicted based on previous knowledge of other members in its protein family. This prior knowledge can define the techniques that will provide valuable information on the allosteric mechanism as well as the transcriptional outcome. Here, we aimed to organize the current structural, dynamic, and functional knowledge of the members of various protein families to provide the reader a framework for the characterization of TRs responsible for new biological outcomes, as well as a previously characterized regulatory network that involves uncharacterized TRs.

In this review, we highlight the state-of-the-art techniques used to elucidate bacterial allosteric TRs’ functions, diversity, and molecular mechanisms. We present a roadmap that serves as a guide to discuss key experiments, based on our initial understanding of the systems studied. This is accompanied by brief descriptions of the advantages and disadvantages of the common approaches, through experimental or bioinformatic techniques. We also present information on a series of databases related to bacterial TRs, alongside a discussion about the available structural information for each protein family. This review closes with a discussion of the multipronged strategies used to study the ensemble models of allostery, as well as the structural and functional characterization in the cellular environment. Taken together, the data obtained from these experiments may provide insight into the highly complex systems that bacteria use to orchestrate transcriptional responses to environmental queues.

## 2. Identifying the Biological Role and the Cognate Inducer

**Figure 1:**
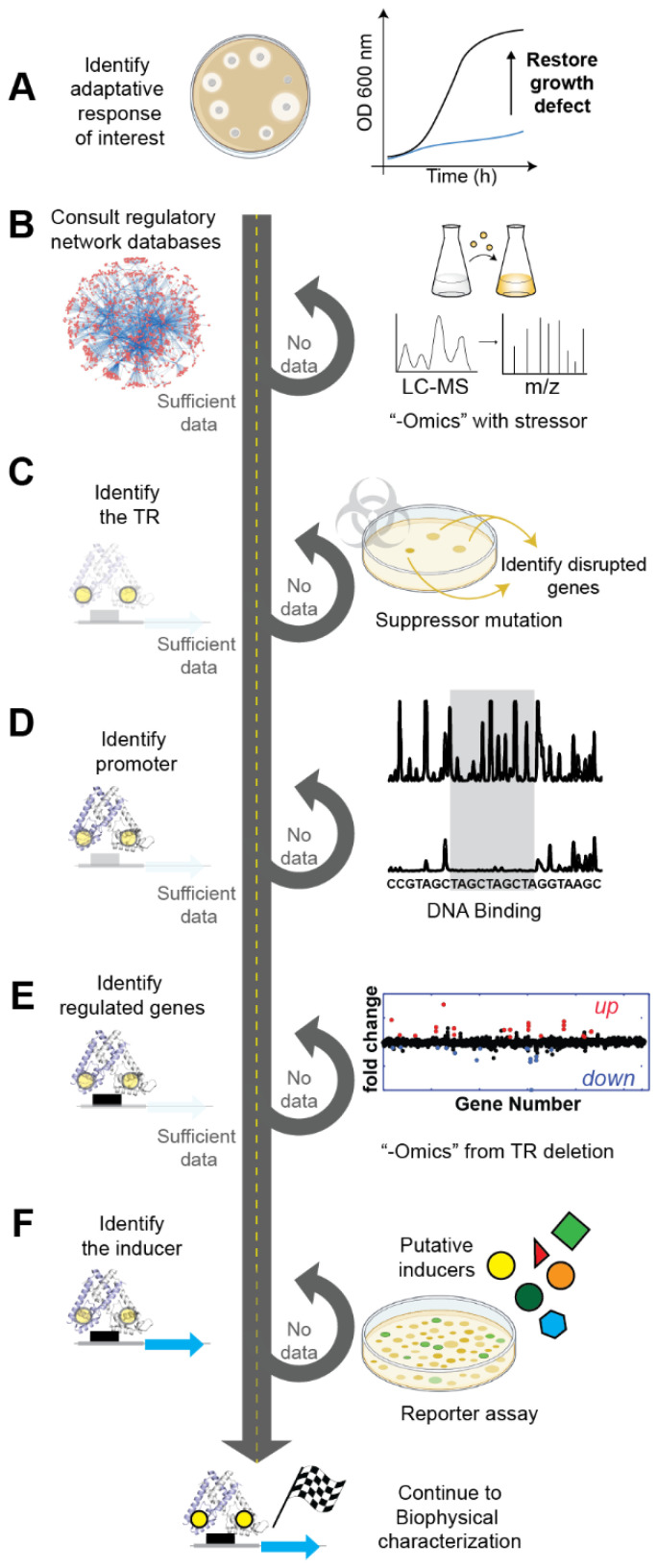
A possible workflow for the biological characterization of an adaptative response. (**A**) Identify the growth defect or resistance mechanism that constitutes the adaptative response of interest. (**B**) Consult the existing databases or employ available bioinformatics tools to collect the necessary data to characterize the components of the regulatory network (i.e., regulator and regulon). If the available information is insufficient or the organism is too novel, transcriptomics, proteomics, and genomic experiments are the most efficient strategy to identify the regulon. (**C**) The existing databases and the “-omics” experiments generally allow for the identification of the TR; when this is not the case, suppressor mutations enable the discovery of the putative TR. (**D**) The identification of the promoter often comes from prior characterization of the regulon; however, it can also be obtained through DNA-binding experiments that can be performed either in vivo or in vitro. (**E**) Some regulated genes are, in most cases, known, as these are the main players in the adaptative response; however, to characterize the complete regulon transcriptomics and proteomics of the deletion are ultimately necessary. (**F**) Once the regulon and the promoter regions are identified, the correct inducer is crucial to allow for further biophysical characterization of the system and can be performed by low through-put reporter assays.

Typically, the main motivation for the characterization of a single-component system is prior knowledge of its participation in a resistance mechanism of interest or adaptative response (i.e., adaptation to a stringent environment, antibiotic resistance, host immune response resistance) (Figure 1A). This prior information is key to defining the experiments that will allow for the identification of the regulon responsible for that adaptative response. The goal was to understand which processes regulate a response that allows for the bacterial adaptation to a stressor molecule. In this section, we discuss the different experimental and computational approaches to identify the regulator, promoter, regulated genes, and inducer.

Bioinformatics is a powerful tool to find genes associated with a function or involved in a regulatory network (Figure 1B, left). In the case of paradigmatic organisms, such as *E. coli*, databases contain the up-to-date description of regulatory networks, including the organization of the genes in transcription units, operons, and entire regulons based on experimental data [17,18,19,20,21]. Additionally, curated databases containing exclusively functional information on TRs [22] or their putative DNA-binding sites [23,24,25] are constantly expanded. Hence, finding the annotated genes corresponding to a TR of interest linked to the regulated genes can be a direct task. In other bacteria, gene annotation is often not as complete; thus, new regulatory networks must be established to find regulons of interest. There is a constant effort to improve regulon prediction with bioinformatics tools [26,27,28] to address the limits set by the degeneracy and variability of the conserved regulatory motifs in operons around their promoter regions. Thanks to this effort, the accuracy of bioinformatic algorithms that combine transcriptomic and other “-omics” data to their pipeline has greatly increased in the last decade [29,30].

When there is not enough data to benefit from bioinformatics, as in the case of pathogens causing neglected diseases [31], “-omics” experiment analyses are generally necessary (Figure 1B, right). A possible starting point is to define which transcription units are affected after exposure to the environmental change of interest, as this type of experiment does not require prior knowledge of the regulatory networks in the organisms, albeit information about the growing conditions and media is required. In recent years, the analysis of the transcriptomic data from prokaryotes has become widely used [32] in the form of RNA-seq [33] and tiling array [34] experiments that are capable of quantifying variations in gene expression with changes in the media. More recent techniques include proteomics experiments to correlate the effects in translation with an observed phenotype [35].

After the transcriptional response is defined, a common strategy is to determine the relevant TR (Figure 1C) and the DNA sequences where it binds (Figure 1D). A widely used technique to find the TR of a gene of interest is suppressive mutations, which consist of treating the cells with a mutagenic agent and sequencing the genome in search of disrupted genes that correlate with the transcription level of the gene of interest [36] (Figure 1C, right). More current and powerful strategies can even allow capturing at the genome-level protein–DNA binding, identifying TRs bound to a particular DNA sequence without necessarily linking them directly to an operon, which can be done later bioinformatically [37,38]. Once a TR has been identified, a variety of techniques allow for the identification of the transcriptional regulatory elements that interact with it, namely the operator (Figure 1D, right). In vitro protein–DNA binding methods include EMSA, protein-binding microarrays (PBMs), chromatin immunoprecipitation-based methods (ChIP), and DNase footprinting assays that can provide detailed information about promoter sequences that contain the operator [39,40]. Nonetheless, these methods can be time consuming if there is no prior knowledge of the promoter sequence. They are not a good approach if the length or degree of conservation of the putative operator sequences impairs a robust bioinformatic search. Higher throughput methods are constantly being developed and usually take advantage of deep sequencing coupled with machine learning algorithms to predict promoter sequences. Some of these techniques may not even require prior knowledge of the TR and are based on in vivo massive parallel reporter assays [41,42,43].

Identifying the complete regulon of a given TR is a key step in the characterization of an adaptative response; its difficulty is often related to how broad its metabolic footprint is (Figure 1E, left). Once the TR has been identified it might be possible to infer the regulon simply by genomic context. If that is not the case, the prior knowledge of the inducer can be used to conduct a set of parallel RNA-seq transcriptomic analyses over WT cells and over mutants where the TR has been deleted (Figure 1E, right). The genes whose transcription levels change significantly could be regulated by the TR (also called transcription factor, TF) under study. This study also provides key information about the repression or activation mechanism, as there are multiple ways in which a transcriptional regulator can act [1]. If the transcription factor functions as a repressor by, for example, occluding access of RNA polymerase (RNAP), the regulated genes will show higher levels of transcripts when comparing the deletion to the wild type (Figure 1E, red dots). On the contrary, if the transcription factor acts as an activator, for example, by binding to a region that is upstream of their target promoters and recruiting the RNAP, the regulated genes will show a lower level of transcripts when comparing the deletion to the wild type (Figure 1E, blue dots). There are many other alternative mechanisms of regulation that are compatible with these two opposite results, and one cannot infer direct interaction of the TR with the promoter just based on the results of RNA-seq analysis. In many cases, indirect regulation has been observed [44]. In others, deletion of the TR can induce a global metabolic change, particularly when it is a global regulator, as was the case in the study by Fur [45]. These issues are often partially addressed by complementing the experiment with parallel RNA-seq analysis in the presence and absence of the cognate inducers, where the result will depend also on the inducer being an allosteric activator or inhibitor. In addition, if the inducer recognition site is known, single-point mutants of the TR-inducer binding site are often a good validation if the results match those in the absence of the inducer. However, even with those complementary experiments, the identity of the regulated genes needs to be verified by in vitro direct DNA binding experiments to rule out the possibility of having the transcriptional profile being affected by the presence of a regulatory network where the TR has a regulatory effect other than direct regulation.

Finally, determining the inducer that triggers a specific transcriptional response, and thus, the observed phenotype, is typically performed in vivo [46] (Figure 1F). A library of putative inducers can be assembled by comparing the growth of a wild-type organism with one lacking the TR of interest in the presence of inducers. Then, transcription of a specific gene is quantified with qRT-PCR, relative to the wild-type organism to a library of putative chemical inducers or by a reporter assay (e.g., β-galactosidase), where the promoter region is genetically engineered in a plasmid upstream a reporter gene (i.e., GPF), such that it regulates its expression. The library of putative inducers is generally small and restricted to what can be inferred by the function of the genes that belong to the regulon. For example, in transition metal homeostasis, the specificity of the transporter genes that are part of the regulon generally coincides with the inducer. However, in many cases, this initial screening for the inducer needs to be complemented by more detailed in vitro experiments that determine the binding constant and/or the chemical modification that results from the presence of the cognate inducer in the TR. There are many examples where the transcriptional response is built by indirectly sensing the chemical inducer; thus, this initial screening will yield results that are ultimately not the cognate inducer but chemical species that give rise to other downstream products in the cellular context. This challenge has been clearly depicted in the characterization of *E. coli* MarR, where the cognate inducer was thought to be a weak binder like salicylic acid [47] until more experiments were performed in vitro and in vivo, and Cu(II) was identified as the cognate inducer [48]. An in-depth study of the binding selectivity of a TR to its inducer and promoter [49] can lead to a more profound understanding of their roles in the cellular response.

## 3. DNA Binding and Inducer Recognition

**Figure 2:**
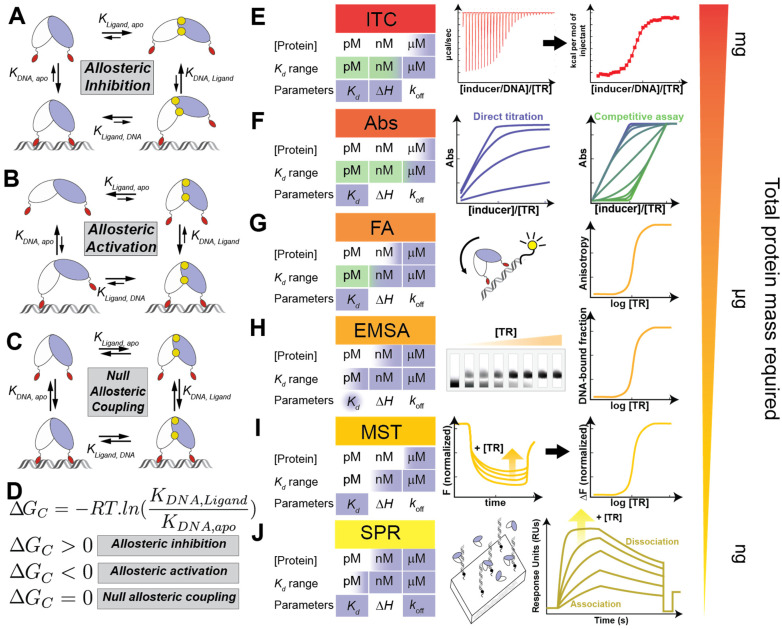
Ligand-binding processes and techniques of interest in transcriptional regulator characterization. (**A**–**D**) General thermodynamic cycles that account for linkage between the four ligation states of a TR, where the TR is represented as a dimer drawing in white and blue with the DNA-binding motif in red (apo, top left; ligand-bound, top right; DNA-bound, bottom left; ternary complex, bottom right). The three possible heterotopic coupling schemes are depicted in each panel—allosteric inhibition (**A**), activation (**B**), and no coupling (**C**). (**D**) The overall coupling free energy (Δ*Gc*, which stands for Δ*G*_*DNA*,*Ligand*_ − Δ*G*_*DNA*,*apo*_) can be determined by measuring either of the pair of equilibrium constants (i.e., vertical or horizontal), and its expression is exemplified by the vertical constant. The allosteric coupling provides insight into the role of a given TR in gene regulatory logic [84]. (**E**–**J**) Summary of the most popular techniques for determining the binding affinities and other parameters for the binding process between the TR and the allosteric ligand or DNA. (**E**) Isothermal titration calorimetry (ITC) is a powerful technique that provides both equilibria and thermodynamic information. Its main limiting factor is the need for mg amounts of protein in high concentrations, around µM, because all measurements are done near the equilibrium (*K_d_*). Direct titration of DNA (blue shading) has a tight binding limit at nM, meaning that equilibria with tighter binding cannot be measured accurately. In competition experiments (green shading), the addition of a competitor that forces the dissociation of the TR–DNA complex allows for the accurate determination of tighter binding equilibrium with *K_d_* even approaching the fM. The different traces (from green to blue) define relative *K_d_s* with respect to the competitor, while in the case of direct titrations, the *K_d_* can be generally modeled by the 50% occupancy of the labeled ligand (blue). ITC measures the heat released by each addition of titrant (TR or DNA). The binding enthalpy is obtained from the total heat released in the first addition, and then the integration of that curve (indicated as a black arrow) provides the function from which the *K_d_* is derived [81,84]. (**F**) Absorption spectroscopy is a practical way to follow a binding event if the system allows it because it does not require special equipment beyond a spectrophotometer. The technique’s drawbacks come from its relatively low sensibility, needing large amounts of protein, and being capable of obtaining only µM *K_d_s* accurately by direct titration (blue traces). Again, the addition of a competitor allows for reaching even tighter binding constants (green traces) with the correct choice of competitor. This is depicted by the change of probe response graphs compatible with extracting a *K_d_* value [74,78]. (**G**) Fluorescence anisotropy (FA) is based on the change of rotational correlation times of differently sized particles; a DNA fragment with a fluorescent probe rotates slower after binding to a protein. Fluorescence experiments have intrinsically higher sensitivity than absorption experiments, requiring smaller amounts of protein and determining tighter binding *K_d_s*. Once more, the addition of a competitor, such as an unmarked DNA fragment, permits the determination of *K_d_s* reaching the fM [62]. (**H**) Electrophoretic mobility shift assay (EMSA) follows the formation of a protein–DNA complex in electrophoresis under native conditions. The accuracy of the determined *K_d_* depends on the correct quantification of the complex formed on the gel, so extracting quantitative information is not an easy task and it is usually correct to confirm it through another of the mentioned techniques. EMSA excels at giving a quick path to observe the formation of the complex, needing low amounts of both DNA and protein. Ethidium bromide (EB) is commonly used to tag the DNA and reach 100 nM *K_d_s*. Fluorescent probes allow for reaching even lower *K_d_s* (around 1 nM), and radiolabeling (32P) is useful for the tighter binding complexes, with *K_d_s* reaching pM [61]. (**I**) Microscale thermophoresis (MST) is based on the differential movement of differently sized/charged particles along a temperature gradient. The normalized signal of a sequence of time traces from tubes containing different concentrations of unlabeled TR in the presence of DNA typically with a fluorescent probe are plotted against the concentration of TR to obtain the *K_d_*. Even if each tube needs a high concentration of protein (µM), the low volume needed means that a relatively small total amount of protein is needed [86]. (**J**) In surface plasmon resonance (SPR), one of the components (usually the DNA) is immobilized on a chip’s surface, while the other component is injected in varying concentrations on the chip’s surface, registering the real-time response. The real-time response allows for the determination of the amount and rate of formation of the complex, giving access to both the binding and kinetic information *k_off_*. SPR usually requires only nanomolar or even picomolar amounts of material, very little compared to other analytical techniques [87]. The protein concentrations were estimated for the experiments where the ligand is labeled, and the protein is in excess with respect to the ligand.

The biological function of a TR that constitutes a single-component system is to elicit a transcriptional outcome in response to a change in the concentration of the cognate inducer. According to this definition, when the inducer modulates the DNA-binding affinity, the function of the TR is defined by the connection between the inducer recognition site and the DNA-binding site, namely the allosteric connection. The biochemical study of binding equilibria in different ligation states allows for the determination of the affinity and selectivity that a particular TR has for its cognate inducer and DNA operator. This ultimately leads to the quantitative determination of the allosteric connection, namely the coupling free energy (Figure 2A–D). This thermodynamic parameter characterizes the function of the transcriptional regulator in charge of responding to exogenous and endogenous stress conditions by inducing gene expression. This framework applies mainly for repressors [50] since, in the case of activators, inducer recognition does not necessarily affect DNA-binding affinity but polymerase recruitment and/or promoter architecture [51,52]. Regardless of the role of allosteric linkage on transcriptional regulation, determining the affinities for each ligand as well as the selectivity is key to gaining a deeper understanding of how bacteria build adaptive responses through TRs. Many TRs function as part of a regulatory network so it is not enough to determine these parameters for a single regulator but for the suite of TRs charged with maintaining cell homeostasis in response to chemical insults from its environment [53,54]. In this section, we introduce the different methodologies that are generally used for describing the different molecular recognition events that define the transcription regulator’s function, namely DNA binding, inducer recognition, and allosteric coupling (Figure 2E–J).

As discussed in Section 2, in many cases beyond the functional characterization in vivo, it is useful to identify the operator region within the promoters of the regulated genes that interact directly with the TR. The benchmark technique to define the actual operator within a promoter is the DNAse footprinting assay [55], which is used to detect the regions of protein–DNA interaction even across the whole genome [56], taking advantage of the fact that a DNA sequence bound to a regulatory protein is protected from nuclease attack relative to flanking exposed nucleotides [57]. DNAse footprinting can provide precise information on the binding site and, in some cases, on the effect of TR binding to promoter conformation. Alternatively, a series of electrophoretic mobility shift assays (EMSAs) can be performed either starting with the whole promoter region or with shorter oligonucleotides that are expected to contain the operator region [58,59]. Prior to DNA incubation, the TR can be treated with the cognate inducer molecule or relevant competing molecules to also test the TR’s specificity [36]. Even though these assays are often used for qualitative purposes, the conditions of these experiments can be optimized and have provided quantitative information on binding stoichiometries or affinities [60,61] of both promoter and inducer binding. This technique provides information about the mechanism underlying the regulation by comparing the affinities of the TR in its apo and inducer-bound state to the DNA and obtaining an estimate of the allosteric coupling (Figure 2H). In cases where the cognate inducer leads to null coupling, the effect of the inducer is not limited to an allosteric effect on the TR, and it is generally linked to the recruitment of other biomolecules, sometimes by solely affecting the DNA geometry (Figure 2C). Another advantage of EMSA is that the quantities needed for the experiments are usually very low, in the range of nM, allowing for numerous experiments. Once the promoter is identified and the preliminary EMSA results are obtained, a more quantitative understanding of the problem is often achieved with other techniques.

In many cases, fluorescence anisotropy (FA) is carried out after or in parallel with EMSA experiments, often giving comparable results [60] (Figure 2G). The main advantage of this is that it is an authentic equilibrium technique that does not involve the separation of the components of the mixture during the measurement, such as the separation that occurs in electrophoresis [62], enabling the study of temperature and salt concentration dependences that inform on the sequence selectivity and affinities in relevant cellular conditions [63]. If FA experiments can be performed for an apoprotein vs. ligand–protein complex, it can ultimately quantify the allosteric regulation in the form of allosteric coupling free energy (Figure 2D). As this technique relies on changes in the hydrodynamic radius of the DNA molecule upon binding to the TF, it is critical that the oligomer has a smaller molecular weight than the TR. So generally, the DNA operator sequence must be determined beforehand to carry out the experiment. On the other side, the experiment is sensitive to protein aggregation; thus, it is not easy to perform with challenging protein complexes [64]. Binding affinities and stoichiometries determined by fluorescence anisotropy have been shown to be useful for modeling actual cellular responses in combination with mass spectrometry-based strategies to accurately determine the intracellular concentration of the TR [52].

Microscale thermophoresis (MST) is a relatively recent and powerful technique that can be used to quantify biomolecular interactions [65,66] (Figure 2I). MST is based on the directed movement of molecules along temperature gradients, an effect termed thermophoresis. Upon the binding of a protein to a DNA or inducer molecule, either the size, charge, and/or hydration shell of a fluorescent molecule within any of the binding partners is changed, resulting in distinct thermophoretic movements of the unbound and bound states. Thus, it has the potential to give essentially identical information to FA experiments, with the main advantage of a minimal requirement of a sample volume (~20 µL) and complex samples, such as plasma and cell lysates [65,67]. MST strongly depends on a variety of molecular properties, which may be advantageous for measuring the binding equilibria associated with minimal molecular changes, such as the ones that may occur on a protein upon inducer recognition. This, however, can also complicate the interpretation, as saturable curves are not always the only result [67]. MST has been extensively applied for drug screening in various proteins, many of them nucleotide-binding proteins [66,68]; however, its application for the characterization of the biophysical properties of allostery in TR remains to be explored more thoroughly. Both FA and MST are reliable techniques to obtain the solution binding constants of TR with DNA, as they minimize artifacts when compared to separation techniques, such as EMSA, or even immobilization techniques, such as surface plasmon resonance (SPR) [69,70,71] (Figure 2J). These low throughput methods all require previous knowledge of the promoter. In recent years, next-generation sequencing techniques have led to methods that can achieve in vitro quantitative determination of affinity together with high binding sequence accuracy by measuring hundreds of thousands of individual binding events as high-throughput insertion tracking by deep sequencing (HiTS) [72].

Depending on the nature of the regulatory mechanism, the inducer recognition may well be as informative as the DNA-binding characterization discussed above. In the case of regulators of metal ion uptake or efflux (namely metalloregulators), a carefully performed but simple colorimetric assay can provide a quantitative measure of the coupling free energy by measuring the metal binding affinities in the presence and absence of DNA [73,74] (Figure 2F). If the metal or ligand of interest induces a spectroscopic change, a direct titration can provide the binding data given that the binding constant is sufficiently low [75]. Alternatively, competition assays can be performed either with non-cognate metals that can replace the cognate inducer [51,76] or with a metal chelator of known affinity [77,78]. These colorimetric assays can provide insight into the regulation mechanism of a TR [79,80] and can be adapted to any system where the inducer provides a spectroscopic signal.

The first step into a more detailed description of the mechanism of an allosteric system is to obtain the thermodynamic parameters that drive the allosteric connection between sites. The benchmark technique for this characterization is isothermal titration calorimetry (ITC), where the heat released after a binding process is measured as the ligand is titrated (Figure 2E). ITC not only allows for the determination of the binding constant but also the enthalpic and entropic contribution in a single experiment. It can be used to determine the cognate ligand of a certain biomolecule and its affinity [81], not only in the case of proteins but also for other one-component systems, such as riboswitches [4,5]. This also allows for quantifying the cooperativity between two inducer binding sites that recognize the same ligand (i.e., homotropic cooperativity) [80]. Of course, that level of detail comes at a cost of the amount of biological material, as it can typically need above micromolar concentration solutions [82], making it expensive and sometimes unpractical to be done if the main question can be answered by a simpler binding assay. The technique is also limited by the range of affinities it can measure (typically between micrometers and millimeters) but it can be extended with competition assays where a moderate affinity ligand is displaced by a high affinity one [83,84]. In combination with other structural techniques, ITC may provide further insight into the thermodynamic parameters of protein binding to DNA, and thus, the mechanism of transcriptional regulation [85].

The abundance of analytical techniques and binding assays available to quantify the affinities of TRs to DNA and inducers allows for choosing based on the system, the information already known, and the question to be answered. In vitro characterization of a novel TR as molecular recognition defines its function. As with all allosteric proteins, the function of TRs cannot be defined by a single-equilibrium process (Figure 2A–D), and to obtain the full characterization of a repressor, we need at least two binding constants (Figure 2D). On the other hand, for activators, we generally need functional in vivo characterization as well as more holistic structural work, as is described in the following sections. Moreover, as TRs are tuned to the intracellular availability of their cognate inducer, the binding assays performed in vitro in diluted solutions of purified proteins should be interpreted as a proxy to understand the protein function rather than a prediction of the biological outcome in the intracellular *milieux* [53].

## 4. Structural Characterization of the Different Allosteric States

Given the importance of TRs in gene regulation and considering the abundance of these “sensor proteins”, structural and functional databases are key for the understanding and prediction of uncharacterized ones. X-ray crystallography as well as high-throughput sequencing methods have allowed for the building of extensive databases of the more than 20 structural classes of so-called one-component transcriptional regulatory systems in prokaryotes. Altogether, the structural and sequence information available has the potential to elucidate key details of the allosteric mechanisms, determine the binding motifs, and more recently, to predict the structure of uncharacterized regulators [7,88]. However, as with any allosteric system, TRs bring an additional challenge, as at least two of the four allosteric states must be described to begin understanding the molecular basis of their function. In this section, we discuss how different structural families of TRs have been interrogated and what is known in terms of the sequence–structure–function in these systems.

The prokaryote TR databases discussed in Section 2 of this review condense most of the structural and functional data available to date. Beyond manually curated databases that contain mostly functional data from a handful of organisms (*Corynebacterium genus* [20], *Bacillus subtilis* [21], *Escherichia coli K-12* [17,19], and *Mycobacterium tuberculosis H37Rv* [20]), for *E. coli*, computational structural prediction methods can be used to infer the putative DNA-binding sites given a TR’s sequence, as implemented in the TF2DNA database [24]. Structural databases that work based on the domain architecture and sequence similarity (such as P2TF [89]) are useful to establish the evolutionary relationships, conservation of residues, and visualization of the genomic context. Alternatively, information about the TR’s structural family can be predicted using homology models based on profile hidden Markov models, as it has been implemented in the DBD transcription factor database [90]. These and other homology models also provide structural predictions for apoprotein states for most TRs. However, these strategies have been clearly surpassed by the introduction of highly accurate deep learning algorithm models, such as AlphaFold [7,91,92]. Beyond the possibility of using databases to identify the protein family and obtain good structural predictions of a single allosteric state [88], these recent developments face different challenges when predicting structural changes upon ligand binding. Thus, to predict the holo form of a TR with its cognate ligand, it may be more insightful to use traditional homology modeling (template-based) using templates that contain the ligand coordinates since more accurate deep learning algorithm models of the apo form would be missing the ligand information entirely. This complication, currently present in the most recent models released by AlphaFold [7,91,92], will likely be solved once user selection of the appropriate ligand-bound template is allowed [93,94] or docking tools are incorporated into deep learning algorithm models [95]. Additionally, sequence similarity networks are a useful tool to provide multiple sequence alignments that would inform better structural predictions, as they have been shown to define isofunctional clusters in TR families [96].

**Figure 3:**
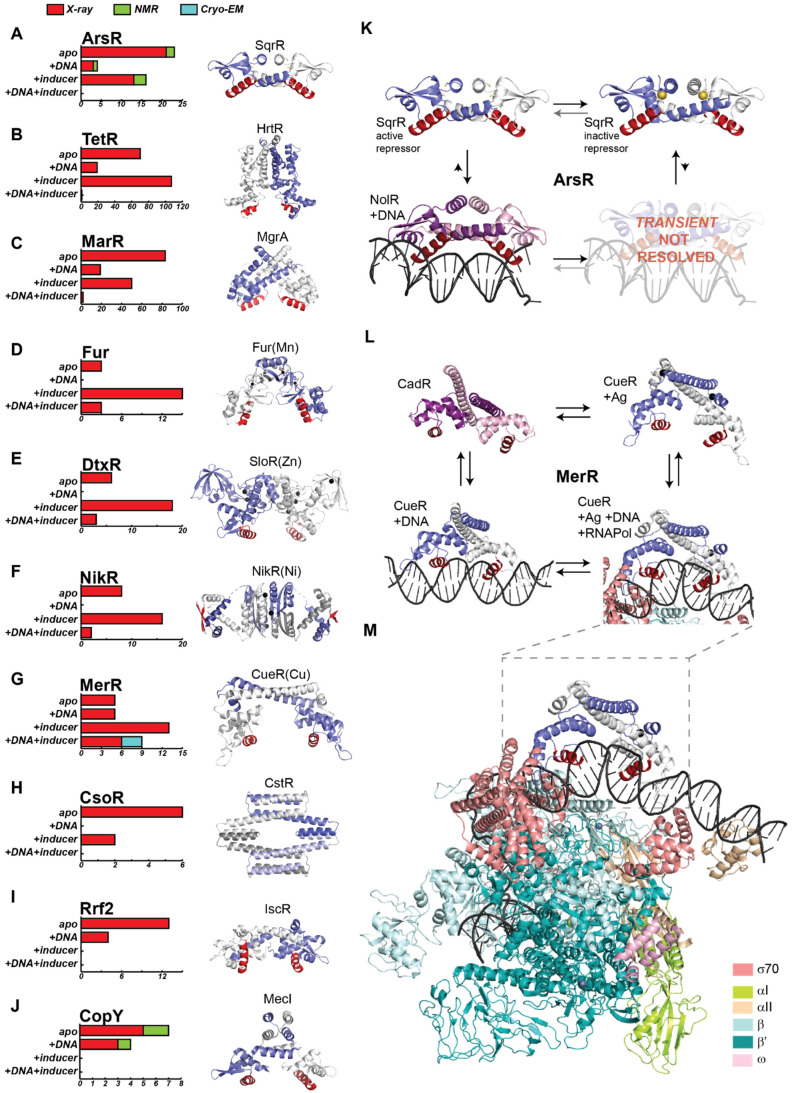
Summary of the available structural information on ten TR families of proteins. (**A**–**J**) Left, bar charts show the total number of protein structures deposited for each protein family in the Protein Data Bank to date, grouped by allosteric state (namely: apoprotein, in complex with DNA, bound to inducers, and ternary protein–DNA–inducer complex). The bars are colored according to the technique used for structural determination (X-ray in red, Cryo-EM in blue, NMR in green). Right, ribbon representations of representative models of each TR family are shown with individual protomers colored white and blue in each case, with the DNA-binding motif shaded red on both protomers. Metal ions are colored black. (**A**) *Rhodobacter capsulatus* C9S apo and reduced SqrR (6o8l) [109], a member of the ArsR family of proteins, which possess a DNA-binding, winged helix–turn–helix domain. (**B**) *Lactococcus lactis* apo HrtR (3vox) [113], a member of the TetR family of homodimeric repressors, in which each subunit harbors an N-terminal three-helix DNA-binding domain, followed by a much larger C-terminal regulatory domain that incorporates a number of independently evolved ligand-binding cavities. (**C**) *Staphylococcus aureus* apo MgrA (2bv6) [114] belongs to the MarR family of TR, which shares the same winged-helical DNA-binding fold of ArsRs but harbors an additional C-terminal helix in the dimer interface, which leads to a more triangular shape. (**D**) *Magnetospirillum gryphiswaldense MSR-1* Fur bound to Mn (II) (4raz) [115] is a member of the Fur family of proteins, and therefore, harbors an N-terminal winged-helix DNA-binding domain, with the regulatory and structural metal-binding sites found in the carboxy domain of the protein. (**E**) *Streptococcus mutans* SloR bound to Zn (II) (5cvi) as a representative member of the DtxR family, with homodimers consisting of an N-terminal winged-helical DNA-binding domain and C-terminal domain harboring often two structurally distinct, yet conserved metal-binding sites per protomer. (**F**) *Escherichia coli* NikR in the Ni (II) form (2hza) [103], a member of the ribbon–helix–helix superfamily of proteins, NikR. (**G**) *Escherichia coli* Cu(I) sensor CueR, bound to inducer (1q05) [116], a model for MerR proteins, which possess an N-terminal winged-helical domain composed of a canonical winged-helix-β hairpin structure, followed by a long dimerization helix that forms an antiparallel coiled coil in the homodimer. (**H**) *Streptococcus pneumoniae* C9A N55A CstR in the reduced state (7mq3) [96] belongs to the CsoR family of TR, characterized by a disc-shaped D2-symmetric or pseudosymmetric tetrameric architecture. (**I**) *Escherichia coli* apo IscR (4hf0) [117], a member of the Rrf2 family of TR, which possesses a DNA-binding, winged helix–turn–helix domain. (**J**) *Staphylococcus aureus* apo MecI (1okr) [118] as a representative member of the CopY family, characterized by a winged helix-like DNA-binding domain. (**K**) ArsR family members, as transcriptional repressors, account for an allosteric inhibition type of thermodynamic cycle (Figure 2A). *Rhodobacter capsulatus* C9S SqrR is resolved both in its apo (reduced) (6o8l) [109] and tetrasulfide (oxidized) (6o8n) [109] forms, but has not been resolved bound to DNA, unlike DNA-bound *Sinorhizobium fredii* NolR (4omy) [119], the individual protomers of which are colored pink and purple, and its DNA-binding motif is shaded dark red, as it is a different structure from SqrR. (**L**) MerR family members, which present null allosteric coupling (Figure 2C), have been resolved in all 4 states of the thermodynamic cycle—*Pseudomonas putida* apo CadR (6jgv) [120] (pink and purple), DNA-bound *Escherichia coli* CueR (4wls) [52], *Escherichia coli* CueR, bound to Ag (I) (1q06) [116], and *Pseudomonas putida* CueR transcription activation complex (7c17) [112]. (**M**) As a ternary protein–DNA–ligand complex, the Cryo-EM structure for the transcription activation complex is shown in detail, comprising *Escherichia coli* CueR, RNAP holoenzyme, and promoter DNA. The color key for the RNAP subunits is presented in the figure.

For many TRs, determining its structure in at least two of the four allosteric states has proven to be a challenge (Figure 3A–J, bar charts from most families are missing at least one state). Particularly when considering that the crystallization of DNA-bound complexes requires the optimization of additional parameters, such as engineering DNA fragments to promote crystallization [97,98], and optimizing the buffer conditions and protein–DNA ratios, as DNA excess may impact the homogeneity [99,100]. Therefore, it is not unexpected that by 2007, there were only three protein–DNA operator complexes deposited from a single protein family [101,102,103] (Figure 3E,F). Since then, considerable progress has been made on structural studies of the bacterial transcription factors, as there has been an improvement in the number of crystal structures of TRs forming a ternary protein–DNA–ligand complex.

An analysis of the structures deposited on the PDB for ten different protein families suggests that the number of structures and the preference for a particular technique for its resolution is not uniform across these families [104] (Figure 3A–J). As expected, X-ray crystallography is by far the most frequently used technique for the structural characterization of proteins, notably surpassing NMR and Cryo-EM in the total number of structures solved by these techniques. It is important to note, however, that the number of structures solved using Cryo-EM has increased exponentially in recent years, and it has made a dramatic change in our understanding of transcriptional activation, as discussed below. While it is true that larger families have more reported structures (for example, there are 44 structures of the 3000-member ArsR family, while there are 198 structures of the 200,000-member TetR family [105]; Figure 3A,B), the number of structures and type of ligation states reported cannot simply be inferred from the number of available sequences. For all the protein families that are transcriptional repressors that release DNA upon inducer recognition, there are no structures reported of the proteins along with its inducer and DNA-binding operator sequence (Figure 3A–C,I,J). This is strictly the case for ArsR proteins (Figure 3A) and there is only one exception *per* family in TetR, and two in MarR, as these are subfamilies that are known to have corepressors [106,107,108], and therefore, the structures of the ternary protein complexes have been solved (Figure 3B,C). Similarly, there are no reported structures of apoprotein–DNA complexes when the ligand is a corepressor acting as an allosteric activator (Figure 3D–F). In the case of CopY family members, the inducer can act as an allosteric activator or as an inhibitor of DNA binding, and the structures are mostly for the apoprotein state of different family members, except for a few structures of the DNA-bound state where only the DNA-binding domain is resolved (Figure 3J). Probably, the only family that has a comparable number of structures solved in each of the four ligation states is MerR, as its complex with DNA is stable in the presence and absence of the inducer (Figure 3G). Finally, newer families, such as CsoR (Figure 3H) and Rrf2 (Figure 3I), have only a few solved structures. The limited information about DNA recognition in CsoR and the changes upon inducer binding in Rrf2 has limited our understanding of these families; thus, the mechanism of allosteric regulation remains speculative.

From these ten protein families, the ArsR family is unique in that full-length structures have been reported by using NMR, which accounts for their high solubility, a particular requisite of this technique (Figure 3A). One remarkable aspect of the ArsR is that the dimer architecture changes upon DNA binding; however, inducer recognition creates only minor structural perturbations [105,109,110] (Figure 3K). These two features have elicited interest in the NMR community, and these proteins have been more extensively looked at in terms of the internal protein dynamics, which are discussed in Section 5.

On the other hand, Cryo-EM has allowed for the determination of large protein–DNA complexes, and at the same time, provides an excellent resolution of these complexes, as it has been recently reported for CueR and BmrR, transcriptional activators from the MerR family [111,112] (Figure 3L,M). One aspect of this family relies on the lack of coupling free energy, meaning that the inducer binding does not affect the affinity for the DNA (Figure 2C and Figure 3L). Instead, inducer binding changes the protein and DNA conformation, eliciting a transcriptional response based on polymerase recruitment [111,112] (Figure 3L). Thus, the unraveling of the structural details of this mechanism of transcriptional activation (Figure 3M) has markedly benefited from the recent advances in structural biology. Changes in DNA topology, such as the ones MerR is known to introduce, are likely more widespread among TRs, and we expect that the resolution revolution from Cryo-EM along with improvement in prediction algorithms will continue to shed light on the structural and functional relationships in these allosteric systems.

## 5. Ensemble Models of Allostery

**Figure 4:**
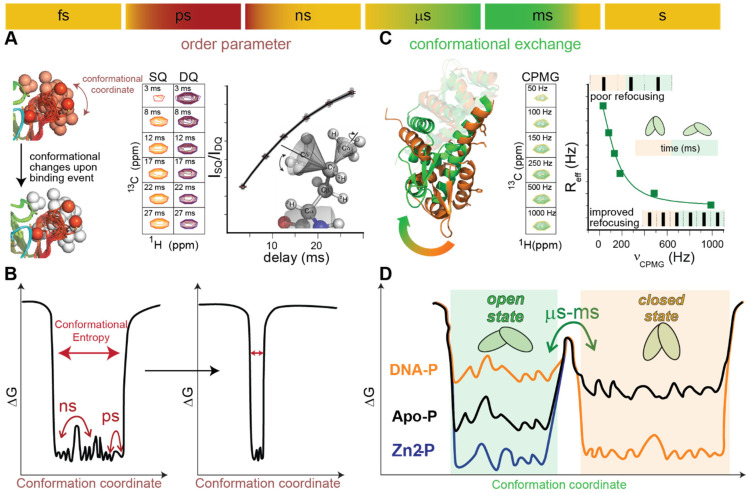
Time scale of the dynamic processes in proteins and the experimental methods that can determine small-amplitude conformational changes in different time scales are represented by the bar on the top. (**A**) ps–ns equilibrium dynamics (shaded red on the time scale bar and illustrated by the protein cartoon on the left) can be studied with relaxation experiments that involve NMR probes that experience forbidden transitions (double quantum, DQ, depicted by purple cross peaks at different delays) performed in parallel to the allowed (single quantum, SQ, depicted by orange cross peaks at different delays). The amplitude of these motions (illustrated by a sidechain drawing on the right panel) is reflected by order parameters obtained from the fit intensity ratio (black trace on the right panel). This ultimately allows for the estimation of conformational entropy changes upon binding, as the sidechain order parameter is a proxy for the number of conformations a protein visits in the sub-nanosecond time scale [152] (left). (**B**) Free energy diagram differences are introduced by a ligand-binding event, such as the one depicted in panel (**A**) (left), which reduces conformational entropy by decreasing the amplitude for a particular coordinate. (**C**) Micro- and millisecond time scale equilibrium dynamics (shaded green on the time scale bar and illustrated by the protein drawing on the left) can be studied with CPMG relaxation dispersion NMR experiments. If the molecule interconverts between two conformational states denoted by open (green) and closed (orange), the intensity of the cross peaks for the NMR reporters will increase their intensity when increasing the frequency of the CPMG pulse (middle panel, cross peaks in green). In these experiments, the dependence of R_2eff_ (proportional to *-ln*(intensity)) with the frequency of pulses directly reports on the degree of chemical exchange of a particular probe, as the protein visits different conformations [153]. (**D**) Free energy diagrams for an allosteric mechanism that relies on conformational selection and can be studied by CPMG based experiments; here, it is exemplified by a simplified version of the CzrA landscape [110]. Apo state shown in black, DNA-bound state shown in orange, and Zn-bound state shown in blue. The apoprotein can visit open and closed conformations in the µs–ms scale, while DNA binding selects the closed conformation, and the allosteric inhibitor selects the DNA incompatible conformation. All the spectra are exemplified with methyl sidechains as reporters, as these are particularly well suited for TR DNA complexes based on their increased sensitivity [153].

Allosteric concepts have departed from structure-based allosteric models by taking advantage of the improvement in experimental technologies to study what is effectively an ensemble of rapidly interconverting configurational states in solution [13]. The problem of allostery in bacterial transcriptional regulators has not been an exception to this process [121]. Recent discoveries emphasize how rigid body movements [122], folded yet dynamic structures [110,123], and disordered regions act to facilitate allosteric connection in TRs [60,115,124]. In this section, we place particular emphasis on the conformational changes that occur in both sub-nanosecond as well as microsecond to millisecond time scales (Figure 4A,B).

The initial motivation for studying the dynamic changes within transcriptional regulators was derived from the extensive conformational changes observed in different ligation states [103,125], such as the ones observed in NikR upon DNA binding, where the two external domains that contain the DNA-binding beta strands bend to fit onto the DNA [103] (Figure 3F). The main goal was to determine if these changes are the result of a preestablished equilibrium or a ligand-induced fit [126]. Most of the early studies were conducted computationally, as capturing conformational transition events of long time scales remains an experimentally challenging task. The main conclusion was that in most cases, ligand binding had a remarkable effect on the conformational spread of the unligated state of the TR, and that the impact on the internal dynamics had a dramatic effect on the DNA-binding affinity and/or complex conformation (Figure 4D, assuming conformational coordinates of larger amplitude). These large-amplitude and long time scale motions are certainly characteristic of multidomain transcriptional regulators [126,127]. In many cases, they are coupled or obscured by changes in the oligomerization state of the protein that is derived from intermolecular interactions between the sensory domains or even the DNA-binding domains [128,129,130]. Overall, when extensive conformational changes occur upon inducer binding, it is still a challenge to obtain direct experimental information about dynamic changes. Generally, multipronged strategies are successful in providing information about the conformational spread of ensembles in a solution [60]. For example, in the case of NikR, a combination of molecular dynamics, small-angle X-ray scattering (SAXS) experiments, and analysis of the available crystal structures validated the initial hypothesis derived from molecular dynamics [122]. A more recent example was derived from the CopY–BlaI family of proteins (Figure 3J), which includes a Cu(I)-sensing metalloregulatory repressor in *S. pneumoniae* that has a unique mechanism of allosteric activation by Zn(II) and allosteric inhibition by the Cu(I) of DNA binding [60]. The SAXS data were combined with EXAFS and NMR data and cysteine reactivity experiments to show that Cu(I) binding has a particular ligand geometry that induces protein aggregation, while Zn(II) binding favors a conformation more compatible with DNA binding. The conformational ensembles of the apo-state, the Zn(II)- and Cu(I)-bound states, were further interrogated with ion mobility–mass spectrometry (IMS-MS), which informs on the changes in the population of conformations with different cross-sections. Ion mobility–mass spectrometry has emerged as a very powerful experimental strategy to interrogate the conformational spread of a particular ligation state in solution, as new ionization strategies are continuously being developed to allow native-like conformations to be stable in the gas phase [131]. Other techniques that are generally used for studying large-amplitude motion changes upon ligand binding are based on performing dynamics experiments on fluorescent or spin labels. Spin labels were successfully introduced in a MerR family protein (Figure 3G), CueR, and double electron–electron resonance experiments confirmed changes in the distance distribution of the probed-upon inducer binding [132]. The more traditional Förster resonance energy transfer (FRET) [133] with fluorescent probes has been used to track DNA binding [134] as well as the conformational ensemble of RNA-based transcriptional regulation (namely riboswitches [135]); however, these have not been popular choices when it comes to the conformational ensemble of bacterial TRs [136,137]. In conclusion, the state-of-the-art for studying large conformational changes in TRs involves multipronged strategies that can validate hypotheses derived from MD with techniques that inform about the conformational spread in solution and not necessarily site-specific details or precise characteristic times of the equilibrium motions.

For many transcriptional factors, the changes inferred from the crystal structure are of shorter amplitude, such as helix reorientation [124,138], or even so minimal that it is hard to infer allosteric mechanisms from the crystal structures of solely two states of the protein [139], thus suggesting that the allosteric mechanism can be better reflected by measuring the equilibrium dynamics in solution. Possibly, the first folded TRs, where it was shown that internal dynamic equilibrium fluctuations in proteins can contribute to allosteric signal transduction, were the tetracycline repressor (TetR) [140] and the transcriptional activator catabolite activator protein (CAP) [141]. The allosteric mechanism of TetR has been studied by characterizing changes in the unfolding process upon single-point mutations as well as ligation to the inducer (namely, tetracycline). The study suggested that tetracycline binding results in a rigidification of the DNA-binding domains into a conformation that is incompatible with DNA binding [140]. This was one of the first experiments that suggested that internal dynamics could play a role in DNA recognition, which contrasted with the well-known mechanisms where ligand-induced folding was critical for correpression [140]. In the case of CAP, the initial structural information [138] was later complemented by NMR experiments that provided the basis of more extensive use of these techniques in determining the dynamically driven allostery (Figure 4). This ultimately led to a mechanism where the allosteric ligand cAMP quenches both slow- and fast-time scale dynamics and activates DNA binding through a mechanism where conformational selection plays a major role [142] (Figure 4D), superimposed onto changes in conformational entropy [123] (Figure 4C).

The power of NMR as the primary experimental tool for interrogating the role of dynamics in allostery, particularly in the sub-nanosecond and sub-millisecond time scales, became clear with these and other earlier studies (Figure 4) [13,123,142]. However, NMR as a structural technique is inherently limited by concentration, protein size, and the existence of highly disordered regions. In this context, the development of new NMR methodologies continues to drive the field of functional dynamics forward, with contributions to new isotope labeling strategies [143] and pulse sequences [144]. The field has taken advantage of a combination of deuteration and TROSY-based [145] triple resonance experiments [146], which has allowed for the measurement of good-quality NMR spectra from other TRs in different ligation states by avoiding fast relaxation. Thus, these technical advances have enabled the departure from initial ideas from TetR proteins and CAP to the study of other families of TRs, which has ultimately led to different hypotheses on the role of internal dynamics in the molecular evolution of allosteric connections [147]. In MarR family proteins (Figure 3C), molecular dynamics [148] and NMR experiments [142] have identified how, on the same molecular scaffold, internal dynamics can be tuned to allow for allosteric inhibition as well as allosteric activation. In this family of proteins, the orientation of the DNA-binding helices is determined by the internal flexibility of the dimer, which is affected by the inducer. In the case of allosteric activation, the inducer leads to a selection of a conformation compatible with DNA binding by restricting the flexibility of an interdomain loop that impairs DNA binding [142], while allosteric inhibition is induced by locking an unfavorable conformation for DNA binding [148] (Figure 4D). These NMR- and MD-based approaches to study low-amplitude changes in internal motions have also been applied to a model system in the ArsR family of proteins (Figure 3A), namely CzrA, a Zn-responsive TR [110]. In this case, it was shown that the inducer (Zn(II)) binding quenched the millisecond time scale dynamics, abrogating conformational exchange and locking the protein in a conformation where it was structurally incompatible with DNA binding. Moreover, upon DNA binding, some sidechains showed more flexibility, with a net increase in the conformational entropy of the protein (Figure 4C). The Zn(II) binding not only abrogates the chemical exchange (Figure 4B) but also eliminates this favorable entropic contribution and increases the surface water degrees of freedom [149], locking the protein in a conformation that is not compatible with DNA binding (Figure 4D). These findings reveal that distinct sites in a protein can communicate with one another exclusively through differences in the relative populations of rotameric states of the sidechains, without the need to invoke a defined molecular pathway or significant structural rearrangements. This hypothesis makes the prediction that a new sensing site could then arise simply by exploiting these delocalized dynamical connections. There is still a lot of technical development enhancing our ability to obtain the appropriate resolution for large complexes between TRs and DNA; one recent example consists of using a labeled protein expressed with equal amounts of 2-^13^C and 3-^13^C pyruvate as carbon sources, facilitating the assignment of sidechains that are critical for studying small-amplitude dynamic changes, such as the ones discussed here [150]. Other strategies of selective labeling involve not only protein production, as having DNA labeled in only some selected nucleotides avoids the complete methylation of the DNA and enables the study of the dynamics of large protein complexes [151].

## 6. Simultaneous Functional and Structural Characterization on Cellular Environments

It has been generally assumed that bacterial TRs perform their function by freely diffusing in the bacterial cytoplasm and that in vitro solution experiments are a minimal but robust model of their function. Although that view is still supported by our ability to model cell behaviors on the parameters obtained from in vitro experiments [53], *in-cell* techniques, such as single-molecule fluorescence, have enabled us to explore how the cells’ crowded environment affects TR function. Thus, today, it is possible to visualize these TRs as individual molecules, performing their function in the native cellular context [154].

An example of the unprecedented mechanistic insights from a single-molecule fluorescence experiment in a cellular context (in this case, smFRET) is the recent work on the Fur-family metalloregulator Zur [155] (Figure 3D). Under zinc-replete conditions, Zur–Zn binds to DNA, repressing the transcription of zinc uptake transporters. Single-molecule tracking (SMT) experiments have allowed for the measurement of Zur mobility in *E. coli* cells, which differentiates freely diffusing TRs from the ones bound to DNA. These experiments revealed that in live *E. coli* cells, Zur’s dissociation rate from DNA is sensitive to the protein concentration in a biphasic manner, initially impeded and then facilitated with increasing Zur concentration. This observation challenges the conventional models of protein dissociation being unimolecular processes (i.e., independent of the concentration) and may explain how bacteria can turn off the transcription of the metal resistance genes by using the intracellular free repressor after metal stress has been relieved. Although this phenomenon could be attributed to high protein concentrations due to the overexpression of the regulator that is otherwise in very low copy numbers, it remains an interesting observation that has been reported in regulators from different families of proteins (Figure 3D,G) [134,156,157].

Recent single-molecule experiments have highlighted the role of compartmentalization in bacteria by membraneless organelles in defining and organizing biochemical functions [158,159,160]; however, our understanding of how these processes affect transcriptional regulation in bacteria is still limited. Post-translational modifications (PTMs) on proteins are known to be one of the described driving forces of biomolecular condensation [161], and many transcriptional regulators are induced by PTMs, so it is likely that condensation can amplify the regulatory signal. Moreover, recent super-resolution imaging experiments on a eukaryotic transcriptional activator [162] suggest that even in the absence of a defined chemical inducer, external stimuli can trigger condensation, PTMs, and activation.

One interesting aspect of the compartmentalization phenomena is related to the observation that these elements are dynamically regulated (by their assembly and disassembly) depending on the environment as they lack a membrane [163]. This dynamic regulation can potentially affect the regulatory response of TRs. Besides the well-known condensates formed by DNA-enhancers and transcription factors [164], there is evidence in eukaryotic cells that supports transcriptional condensates derived from emergent RNAs [165]. This provides dynamic feedback through its RNA product, as these condensates are dissolved after an excess of RNA is produced. Although there is no evidence of these kinds of processes in bacteria, the ubiquity of phase separation, especially in distantly related organisms, suggests that this may be an evolutionarily conserved mechanism [166]. Thus, it is interesting to consider the impact of the observed membraneless organelles in transcription, as they could be affecting transcription reaction rates by spatially regulating diffusion-controlled binding processes with DNA [167,168]. This is an exciting direction of future research, as the limit of microscopy techniques to identify these condensates has significantly improved in recent years, enabling us to find them even in bacteria where the size is usually close to the diffraction limit [159,169,170]. This is particularly relevant for bacterial transcriptional regulation, as many of these proteins contain intrinsically disordered regions IDRs [105,115,124,171,172] (Figure 3A,C,D,H–J), which is a common feature of proteins included in condensates [173,174,175].

Confirming the possible relevant role of these processes beyond eukaryotic cells, it is the case that *E. coli* RNA polymerase can form these biomolecular condensates or clusters by itself, enabling the regulation of nutrient-dependent transcription [176]. Fluorescence imaging and single-molecule tracking experiments show that RNAP is distributed throughout the nucleoid in cells grown in minimal media, while it concentrates into distinct clusters when cells are grown in rich media. These weak protein–protein interactions between RNA polymerase proteins have also been described in other bacteria, giving rise to cluster formation and not necessarily condensates [177].

Observing condensates in bacteria will continue to benefit from the advances in super-resolution microscopy and other techniques [178], as they are significantly smaller compared to eukaryotic condensates [169]. Recently developed photoactivated localization microscopy (PALM) and stochastic optical reconstruction microscopy (STORM), as well as cryogenic super-resolution imaging, are valuable tools to evaluate condensates in bacteria [159,179]. We expect that this will be an area of exciting research in the following years, aiming to contribute to a more holistic understanding of transcriptional regulation in bacteria by combining their insights with multipronged strategies, as presented here.

## 7. Conclusions and Future Perspectives

Bacterial survival requires swift responses to various adverse stress conditions. As discussed in other work published in this issue [180,181,182], transcriptional regulation is key for this adaptation. In this review, we focused on bacterial, allosteric TRs, highlighting the state-of-the-art techniques used for functional, biophysical, structural, and dynamic characterization. We expect that it will be helpful to not only researchers that focus on the characterization of a particular transcriptional response but also to an emerging community of researchers interested in putting what is known about a particular TR protein or family into perspective. There is a diverse community interested in these systems, from microbiologists interested in finding new targets for possible antimicrobial therapies [183] to synthetic biologists aiming to identify new regulators to incorporate into devices [184].

Future areas of exciting research in the field will likely emerge from bioinformatic analysis, taking advantage of the wealth of structural, functional, and “-omics” data on TRs, and paving our understanding of the evolution of bacterial transcriptional responses. We believe that the evolution of regulation and antibiotic resistance defines a thrilling field of research, as protein dynamics and allostery have likely facilitated the rapid rise of new functionalities in bacterially encoded regulatory proteins. As bacterial resistance becomes a major threat, understanding key aspects of how bacteria respond to stress conditions by regulating biological outcomes will play a critical role in preventing deadly pandemics in the post-antibiotic era.

## Data Availability

Not applicable.
